# Spironolactone Induces Vasodilation by Endothelium-Dependent Mechanisms Involving NO and by Endothelium-Independent Mechanisms Blocking Ca^2+^ Channels

**DOI:** 10.3390/jox14010020

**Published:** 2024-03-01

**Authors:** Margarida Lorigo, João Amaro, Elisa Cairrao

**Affiliations:** 1 CICS-UBI, Health Sciences Research Centre, University of Beira Interior, 6200-506 Covilhã, Portugal; margarida.lorigo@ubi.pt (M.L.); joao.costa.amaro@ubi.pt (J.A.); 2 FCS-UBI, Faculty of Health Sciences, University of Beira Interior, 6200-506 Covilhã, Portugal

**Keywords:** mineralocorticoid receptor antagonist, cardiovascular, endocrine-disrupting chemical, rat aorta, pharmacology, vasodilation

## Abstract

Background: Spironolactone (SPI) is a diuretic widely used to treat cardiovascular diseases (CVD) and is non-specific for mineralocorticoid receptors (MR) and with an affinity for progesterone (PR) and androgen (AR) receptors. Since 2009, it has been suggested that pharmaceuticals are emerging contaminants (called EDC), and recently, it was reported that most EDC are AR and MR antagonists and estrogen receptors (ER) agonists. Concerning SPI, endocrine-disrupting effects were observed in female western mosquitofish, but there are still no data regarding the SPI effects as a possible human EDC. Methods: In this work, aortic rings were used to analyze the contractility effects of SPI and the mode of action concerning the involvement of Ca^2+^ channels and endothelial pathways. Moreover, cytotoxic effects were analyzed by MTT assays. Results: SPI induces vasodilation in the rat aorta by endothelium-dependent mechanisms involving NO and by endothelium-independent mechanisms blocking Ca^2+^ channels. Moreover, a non-monotonic effect characteristic of EDC was observed for SPI-induced decrease in cell viability. Conclusions: Our findings suggest that SPI may act as an EDC at a human level. However, ex vivo studies with human arteries should be carried out to better understand this drug’s implications for human health and future generations.

## 1. Introduction

Cardiovascular diseases (CVD) represent a significant global burden, being a leading cause of mortality and mobility worldwide [1]. In recent decades, rodent models have become commonplace in CVD research [2,3,4]. Indeed, animal models provide valuable insights into vascular function by investigating both endothelial and smooth muscle pathways, thereby enhancing our understanding of CVD pathogenesis, particularly in the context of endothelial dysfunction frequently associated with CVD development. On the other hand, preventing and treating these diseases is quite challenging since certain promising drugs may have opposite effects to the desired ones, i.e., they may promote CVD [5,6,7]. Overall, it seems that certain classes of drugs may be effective in preventing CVD. However, through their different modes of action, these drugs may increase several adverse cardiovascular effects, as with some diuretics, like spironolactone (K^+^-sparing diuretic). Therefore, explaining these phenomena is a significant challenge concerning CVD.

Spironolactone (SPI), a pharmaceutical drug, was the pioneering mineralocorticoid receptor (MR) antagonist developed for the treatment of hypertension, primary hyperaldosteronism, and peripheral oedema associated with heart failure, as well as other aldosteronism-associated conditions. In addition to being a potassium-sparing diuretic, SPI effectively treats progressive heart failure and is quite safe and protective against hypertension, particularly in patients with so-called resistant hypertension [8]. Structurally, SPI is a synthetic derivative of progesterone, which is also a potent MR antagonist. However, both share an agonist activity with the progesterone receptor, and SPI is also an androgen receptor antagonist [9]. Consequently, this association presents side effects for females (menstrual irregularities) and males (sexual dysfunction with painful gynecomastia) [10].

On the other hand, since 2009, it has been suggested that pharmaceuticals are emerging contaminants [11]. Acting as endocrine disruptors, these compounds can interfere with hormonal actions by different rather complex and specific molecular and cellular pathways and consequently cause effects on the human cardiovascular system [12,13]. The endocrine disrupting effect for SPI has already been reported in female western mosquitofish, *Gambusia affinis*. In addition, the authors demonstrated that SPI has androgenic and/or antiestrogenic activity [14]. However, there are still no data regarding the effects of SPI as a human EDC. On the other hand, it was recently reported that most EDCs are antagonists of the AR and MR and agonists of the ER [15]. Thus, this work aimed to assess SPI’s vascular effects on the smooth muscle of rat aortas to elucidate whether SPI could potentially function as an EDC in humans. To achieve this purpose, endothelium-intact and -denuded aortic rings were employed to evaluate the contractility effects of SPI using the organ bath technique. In addition, SPI’s vascular mode of action was investigated in-depth, focusing on its involvement with Ca^2+^ channels and endothelial pathways. Moreover, the potential cytotoxic effects of SPI were analyzed through MTT assays.

## 2. Materials and Methods

### 2.1. Ethics Considerations

The research procedures conducted in this study were according to the Animal Care guidelines approved by the Institutional Review Board of the University of Beira Interior under the authorization number T0023 (22 March 2022, Covilhã, Portugal). Furthermore, all experiments were performed in accordance with Directive 2010/63/EU of the European Parliament, which outlines the protection of animals used for scientific purposes.

### 2.2. Experimental Model

This work used 34 male Wistar rats (Charles-River, Barcelona, Spain) aged 3–4 months and weighing 400–500 g. These animals were housed in the animal facilities of CICS-UBI, which are licensed by the Competent National Authority “Direcção-Geral da Alimentação e Veterinária” (DGAV). The rats were maintained under controlled conditions, including a 12 h light/dark cycle and suitable acclimatization conditions. The rats were allowed free access to food and water ad libitum.

### 2.3. Isolation of Rat Aortic Rings

To prepare isolated rat aortic rings, the animals were first euthanized using carbon dioxide (CO_2_) for 2 min. The thoracotomy procedure was carefully performed following the methodology outlined by Lorigo et al. (2022) [16]. The thoracic aorta was promptly dissected, ensuring the removal of surrounding connective tissue and fat, and subsequently cut into small rings measuring 3–5 mm in length. In the case of endothelium-denuded aorta rings, the endothelial layer was mechanically eliminated using a cotton swab inserted into the lumen of the vessel ring. The aortic rings were then mounted in the organ bath chambers (LE01.004, Letica, Madrid, Spain), as previously described by our research group [16]. Subsequently, the aortic rings were submerged in a 10 mL of Krebs-bicarbonate solution containing (mmol/L) KCl 5.0, EDTA 0.03, MgSO_4_∙7H_2_O 1.2, KH_2_PO_4_ 1.2, ascorbic acid 0.6, NaCl 119, CaCl_2_ 1.5, glucose 11, and NaHCO_3_ 25. The solution was adjusted to a pH of 7.4 and maintained at a temperature of 37 °C. The carbogen mixture, composed of 95% O_2_ and 5% CO_2_, was continuously bubbled into the solution. A resting tension of 20–25 mN was applied on each ring, allowing them to equilibrate for 45 min. Isometric tensions were monitored using the method described by Lorigo and Cairrao (2022). Functional removal or endothelial integrity was always analyzed before starting the organ bath experiments by applying 1 μmol/L acetylcholine (ACh) to the arteries. After a stable contractile response to 1 μmol/L PE, the absence of a relaxing response to ACh indicated successful endothelium removal. In the case of endothelial integrity, the rings showed an almost total relaxing response to ACh (90–100%). This assessment considered that 100% relaxation re-established the basal tension value. Therefore, and following previous studies [16,17,18], whenever the relaxation induced by ACh was <10% or >90%, rings were considered “denuded endothelium” or “intact endothelium”, respectively. For each experiment, 4 vessel segments were obtained from each animal.

### 2.4. Ex Vivo Experiments

#### 2.4.1. Vasorelaxant Effect of Spironolactone on Isolated Rat Aorta

The vascular effects of spironolactone were examined on intact- and denuded-endothelium rings pre-contracted with noradrenaline (NA, 1 μmol/L), phenylephrine (PE, 1 μmol/L), or potassium chloride (KCl, 60 mmol/L). Once the aorta rings reached a stable contraction, cumulative concentrations of spironolactone ranging from 0.001 to 100 μmol/L were administered. Control experiments employed ethanol as the solvent at the correspondent % of spironolactone.

The vasoactive agents NA and PE are two agonists of the adrenergic receptors, promoting the activation of different intracellular signaling pathways. The NA was chosen due to it is a potent α1- (coupled to Gq protein), α2-(coupled to Gi protein), and β1- and β2-adrenoceptors (coupled to Gs protein) agonist, while PE is a selective agonist for α1- adrenoceptors (coupled to Gq protein). Concerning KCl, this isosmotic solution was based on KCl (due to the K^+^ increase), a depolarizing agent of the cell membrane. Consequently, it promotes the opening of L-type Ca^2+^ channels and increases the influx of Ca^2+^ into the cell. This differentiated choice allowed us to analyze different vascular outcomes induced by SPI in the rat aorta.

#### 2.4.2. Effects of Spironolactone-Induced Relaxation via L-Type Ca^2+^ Channels (LTCC)

The involvement of L-type Ca^2+^ channels (LTCC) in the spironolactone-induced relaxation was assessed on intact- and denuded-endothelium rings pre-contracted with NA (1 μmol/L), PE (1 μmol/L), or KCl (60 mmol/L). To investigate whether the inhibition of LTCC is implicated in the relaxation caused by spironolactone, a specific LTCC inhibitor, nifedipine, at concentrations of Nif, 0.01, and 1 μmol/L was employed. Subsequently, the effects of spironolactone-induced relaxation were analyzed. Control experiments used ethanol as the solvent at the correspondent % of spironolactone. These experiments were conducted without UV light due to the potential photodegradation of nifedipine.

#### 2.4.3. Effects of Spironolactone-Induced Relaxation via Endothelium

The involvement of the endothelium in the spironolactone-induced relaxation was evaluated on intact-endothelium rings pre-contracted with NA (1 μmol/L) or PE (1 μmol/L). The roles of cyclooxygenase (COX) and nitric oxide endothelial synthase (eNOS) were investigated by treating the rings with specific inhibitors, namely indomethacin (10 µmol/L) and L-NAME (100 µmol/L), respectively. Subsequently, the effects of spironolactone-induced relaxation were analyzed. These experiments were conducted without UV light due to the photodegradation potential of indomethacin.

### 2.5. Smooth Muscle Cell (SMC) Culture

A cell model of vascular smooth muscle cells (SMCs) was selected, specifically, the A7r5 cell line derived from the embryonic thoracic aorta of *Rattus novergicus* (Sigma-Aldrich, Lisboa, Portugal). Cells were cultured in 25 cm^2^ culture flasks using a medium consisting of DMEM-F12 (Sigma-Aldrich, Lisboa, Portugal) supplemented with 1.2 μg/L NaHCO_3_, 20 mg/L L-ascorbic acid (Sigma-Aldrich), 0.25% BSA (bovine serum albumin; Sigma-Aldrich), 10% FBS (heat-inactivated fetal bovine serum; Biochrom, Cambridge, UK), and a mixture of antibiotics (5 U/mL penicillin, 5 μg/mL streptomycin, and 12.5 ng/mL amphotericin B (Sigma-Aldrich), as described in previous studies [16,18]. Cell cultures were maintained at 37 °C in a humidified incubator with an atmosphere of 95% room air and 5% CO_2_. The culture medium was refreshed every 2–3 days. When the cells reached approximately 80% confluence, SMC were detached with trypsin incubation (commercial solution (0.3%) in a Ca^2+^-Mg^2+^-free phosphate-buffered solution containing EDTA (0.025%)) and further planted in 96-well plates for cytotoxicity assays.

### 2.6. SMC Cytotoxicity Assay

Cytotoxicity assays using A7r5 cells were performed following the methodology described by Baptista et al. (2022) [18]. The viability of the cells, as a testing endpoint of cytotoxicity, was measured using 3-(4,5-dimethylthiazol-2-yl)-2,5-diphenyltetrazolium bromide (MTT). This assay measures the conversion of a substrate (tetrazolium salt—MTT) into a chromogenic product (purple formazan) precipitated by succinate dehydrogenase from intact mitochondria of living cells. Thus, it enables the evaluation of cell viability and proliferation in vitro in response to an external factor, as formazan production indicates cell viability. The intensity of the purple color, which correlates with the number of viable cells, can be measured spectrophotometrically after diluting the crystals with dimethyl sulfoxide (DMSO).

A7r5 cells were seeded into 96-well plates and incubated for 48 h with spironolactone at concentrations ranging from 0.0001 to 1000 μmol/L. As positive and negative controls, cells were treated with a medium containing 0.01% ethanol and A7r5 cells in a complete culture medium only, respectively. At the end of the incubation period, the treatment medium was replaced with 100 μL of culture medium containing MTT solution MTT solution (0.5 mg/mL) and incubated for an additional 4 h at 37 °C in a humidified incubator (95% room air and 5% CO_2_ atmosphere). Afterward, the MTT solution was removed, and the purple formazan crystals were dissolved in 200 μL of DMSO solution. After the dissolution of formazan crystals, absorbance was determined spectrophotometrically at 570 nm using a Microplate Reader (EZ Read 400, Microplate Reader, Biochrom). Cell viability was calculated using the following formula: (%) = [100 × (sample abs)/(control abs)].

### 2.7. Drugs and Chemicals

All drugs were obtained from Sigma-Aldrich Química (Sintra, Portugal). Chemicals were dissolved in distilled water, except indomethacin and nifedipine, which were dissolved in absolute ethanol. Appropriate dilutions were prepared daily using Krebs solution (for contractility experiments) or medium without FBS (for cytotoxicity experiments). The final concentration of solvent ethanol never exceeded 0.01%.

### 2.8. Data and Statistical Analysis

The data obtained in this investigation were presented as mean ± standard deviation (S.D.). Statistical analysis was performed using Software SigmaStat v3.5, 2006. Two-way ANOVA with interaction followed by Bonferroni’s *t*-tests were used for data analysis, except for the MTT assay data, which were analyzed using one-way ANOVA followed by Dunnett’s post hoc tests. Statistical significance was considered when the *p*-value was less than 0.05. The data and statistical analysis comply with the recommendations on experimental design and analysis in pharmacology.

## 3. Results

### 3.1. Cytotoxicity of Smooth Muscle Cells Induced by SPI

**Figure 1:**
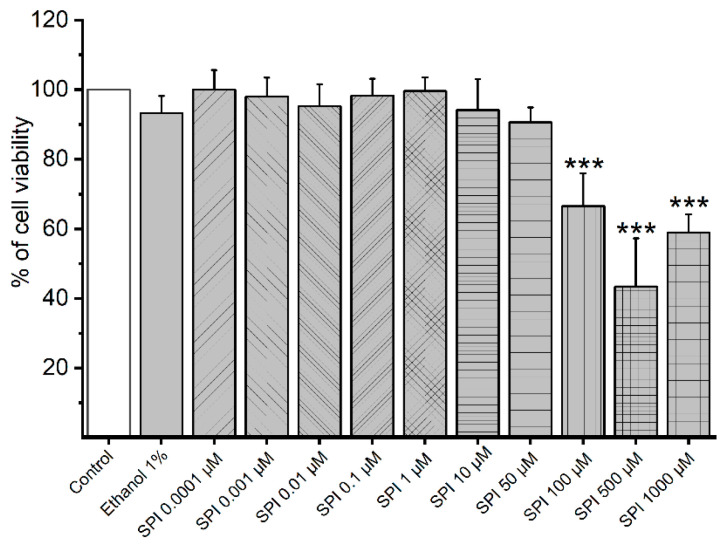
Cell viability of A7r5 cells under the effect of SPI (spironolactone, 0.0001–1000 μmol/L) (n = 12). Data are presented as a percentage (%) of sample absorbance on absorbances induced by control. Each bar represents mean values and vertical lines the S.D. of the mean. * (asterisk) represents differences between SPI and vehicle (ethanol 1%) (*** *p* < 0.001) (*p* < 0.05, one-way ANOVA followed by Dunnett’s post hoc tests).

To investigate cell viability of A7r5 cells under the effect of SPI, MTT assay was used. Figure 1 presents the % effects of different SPI concentrations in the cell viability. The results show that the three highest SPI concentrations (100, 500, and 1000 μmol/L) significantly reduced cell viability (67%, 43%, and 59%, respectively).

### 3.2. Contractility Experiments on Rat Aorta Smooth Muscle

Before the experiments, we analyzed spironolactone’s effects on the basal tension of the rat aortas. Our analysis revealed that SPI did not alter their basal tonus (*p* = 0.336, without endothelium and *p* = 0.529, with endothelium; one-way ANOVA). Subsequently, we initiated the first stage of these experiments to investigate the effects of spironolactone on NA-, PE-, and KCl-contracted aortic rings. The tensions generated by the contractions induced by these three contractile agents used in both endothelium-intact and -denuded rings are presented in Table 1. The results demonstrate an interaction between the contractile agent’s effects and the endothelium’s presence (*p* = 0.012, two-way ANOVA followed by Bonferroni’s *t*-tests). The contractions induced by NA were higher than PE in endothelium-denuded rings (*p* = 0.036). Moreover, a higher contraction induced by KCl was observed compared to the NA and PE (*p* = 0.003 and *p* = 0.004, respectively) in endothelium-intact rings. Comparing the endothelium-denuded and endothelium-intact rings, the contractions induced by NA were higher in endothelium-denuded rings (*p* < 0.001).

### 3.3. Vasodilation Induced by SPI through Ca^2+^ Channels

**Figure 2:**
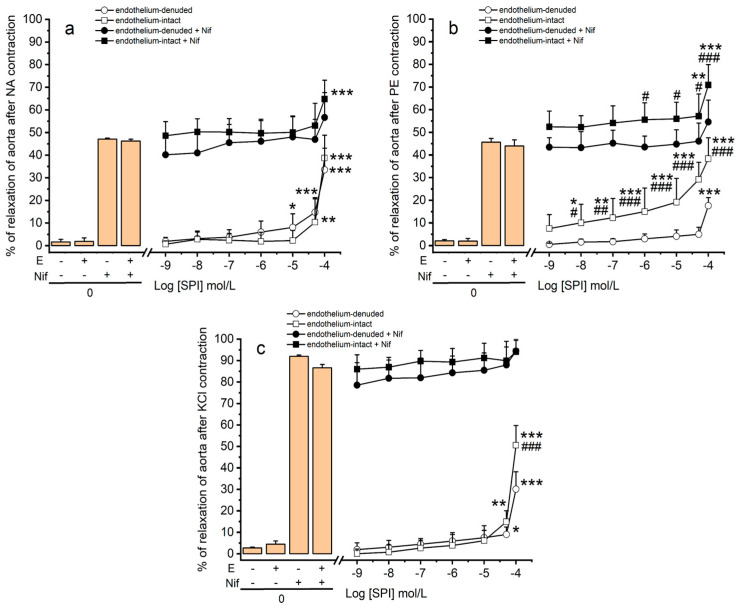
Vasorelaxant effects induced by Nif (nifedipine, 1 and 0.01 µmol/L) plus SPI (spironolactone, 0.001–100 μmol/L) on endothelium (E)-intact and -denuded aorta rings pre-contracted with (**a**) NA (noradrenaline, 1 μmol/L, n = 16), (**b**) PE (phenylephrine, 1 μmol/L, n = 10), and (**c**) KCl (potassium chloride, 60 mmol/L, n = 10). Data are presented as a percentage (%) of relaxation on maximal contraction induced by vasoactive agents. Each bar (left panel) or point (right panel) represents mean values and vertical lines the S.D. of the mean. * (asterisk) represents differences versus control (* *p* < 0.05, ** *p* < 0.01, and *** *p* < 0.001), and ^#^ (cardinal) shows significant differences between endothelium-denuded versus endothelium-intact (^#^ *p* < 0.05, ^##^ *p* < 0.01, and ^###^ *p* < 0.001) (*p* < 0.05, two-way ANOVA with interaction followed by Bonferroni’s post hoc *t*-tests).

The vascular effects of SPI in endothelium-intact and -denuded rings contracted with NA, PE, and KCl 60 mmol/L were evaluated. The results reveal an interaction between of the concentration of SPI and the presence of endothelium (*p* ≤ 0.001, two-way ANOVA with interaction followed by Bonferroni’s post hoc tests). As shown in Figure 2, cumulative concentrations of SPI elicited a vasorelaxation in both groups of aortic rings (endothelium-intact and endothelium-denuded), with a more pronounced effect observed in endothelium-intact aorta rings compared to endothelium-denuded rings (NA << KCl < PE). In endothelium-intact rings, all concentrations of SPI (except the lowest concentration, 0.001 μmol/L) significantly induced vasodilation in PE-contractions compared to the endothelium-denuded rings (*p* < 0.01). Moreover, vasodilation induced by the highest concentration of SPI (100 μmol/L) was more prominent in KCl-contraction in endothelium-intact rings compared to endothelium-denuded rings (*p* < 0.01). In contrast, no significant differences were observed in NA-contractions (*p* > 0.05).

In addition, experiments with a sensitive organic Ca^2+^ channel blocker, nifedipine, were performed to investigate whether the Ca^2+^ channels are involved in the molecular mechanism through which SPI induces its activity. The results demonstrate an interaction between the SPI concentration and the endothelium’s presence (*p* ≤ 0.001, two-way ANOVA with interaction followed by Bonferroni’s post hoc tests). As shown in Figure 2, a vasorelaxation was observed in both groups of aortic rings (endothelium-intact and endothelium-denuded) following nifedipine (Nif) application. A concentration of 1 μmol/L of Nif induced close to 100% relaxation in both endothelium-intact and -denuded KCl-contracted aorta rings [16]. In this sense, a lower concentration of nifedipine (0.01 μmol/L) was used for analyzing the effect of Nif and SPI joint application. The results indicate that the vasodilation induced by the joint application of Nif and SPI was similar to the vasodilation induced by Nif in NA- and KCl-contracted endothelium-intact and -denuded aorta rings (Figure 2a,c, respectively) (*p* > 0.05, two-way ANOVA). In contrast, in endothelium-intact rings contracted by PE, a higher vasodilation was observed (*p* < 0.05 and *p* < 0.001, and a two-way ANOVA with interaction followed by Bonferroni’s post hoc tests) for concentrations of SPI ranging from 1 to 100 μmol/L (Figure 2b).

### 3.4. Vasodilation Induced by SPI through Endothelial Mediators

**Figure 3:**
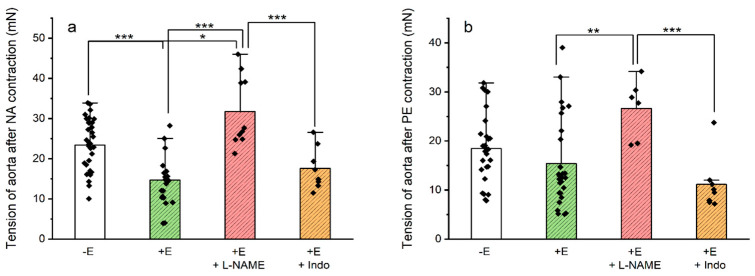
Maximum tensions produced by (**a**) NA (noradrenaline, 1 μmol/L, n = 28) and (**b**) PE (phenylephrine, 1 μmol/L, n = 24) on endothelium-denuded and -intact rings and in the presence of the inhibitors L-NAME (N(ω)-nitro-L-arginine methyl ester, an eNOS inhibitor, 100 µmol/L) and Indo (indomethacin, a non-selective COX inhibitor, 10 µmol/L). The values are expressed as tension (mean) ± S.D. (mN). Dispersion points represent each value. * (asterisk) represent statistical differences between conditions tested (* *p* < 0.05, ** *p* < 0.01, and *** *p* < 0.001); two-way ANOVA followed by Bonferroni’s *t*-tests).

To investigate the molecular mechanism through which SPI induces its endothelial activity, the next step was to use Indo (indomethacin, a non-selective COX inhibitor) and L-NAME (N(ω)-nitro-L-arginine methyl ester, an eNOS inhibitor. The tensions generated by NA and PE in the presence of two inhibitors (L-NAME and indo) on endothelium-intact aortic rings are presented in Figure 3. The results demonstrate that both NA- and PE-contractions in the presence of L-NAME were significantly higher compared to contractions in endothelium-intact rings (*p* ≤ 0.001 and *p* ≤ 0.004, respectively). In contrast, no significant differences were observed when indomethacin was present for the two contractile agents used (*p* = 1.000, NA and *p* = 0.848, PE; two-way ANOVA followed by Bonferroni’s *t*-tests).

**Figure 4:**
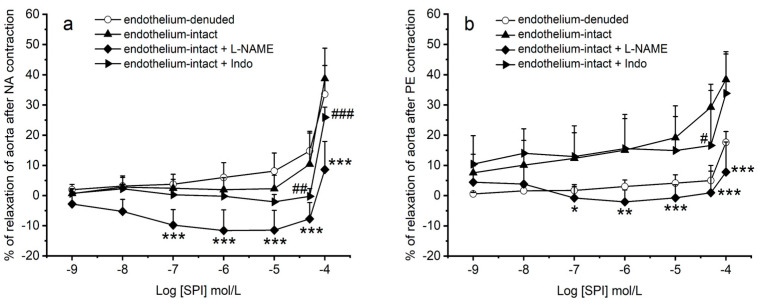
Vasorelaxant effects induced by SPI (spironolactone, 0.001–100 μmol/L) on endothelium-intact and -denuded aorta rings pre-contracted with (**a**) NA (noradrenaline, 1 μmol/L, n = 6) and (**b**) PE (phenylephrine, 1 μmol/L, n = 5) and in the presence of the inhibitors L-NAME (N(ω)-nitro-L-arginine methyl ester, an eNOS inhibitor, 100 µmol/L) and Indo (indomethacin, a non-selective COX inhibitor, 10 µmol/L). Data are presented as a percentage (%) of relaxation on maximal contraction induced by vasoactive agents. Each point represents mean values, and vertical lines are the S.D. of the mean. * (asterisk) represents differences between SPI vs. L-NAME + SPI in endothelium-intact rings (* *p* < 0.05, ** *p* < 0.01 and *** *p* < 0.001), and ^#^ (cardinal) shows significant differences between SPI vs. Indo + SPI in endothelium-intact rings (^#^ *p* < 0.05; ^##^ *p* < 0.01; ^###^ *p* < 0.001), (*p* < 0.05, two-way ANOVA with interaction followed by Bonferroni’s post hoc *t*-tests).

Figure 4 illustrates the effects of spironolactone on endothelium-intact rings contracted with NA and PE, in the presence of the inhibitors L-NAME and Indo. In the presence of L-NAME inhibitor, SPI induced a vascular contraction of NA-contracted aorta rings (Figure 4a) and failed to promote vasodilation in PE-contracted aorta rings (Figure 4b). These effects were observed for all concentrations of SPI, except the two lowest concentrations (0.001 and 0.01 μmol/L) (*p* < 0.01, two-way ANOVA with interaction followed by Bonferroni’s post hoc *t*-tests). Conversely, in the presence of Indo inhibitor, the results demonstrate a decrease in SPI-induced vasodilation in NA- and PE-contracted rings (50 and 100 μmol/L) (Figure 4a,b). An interaction between the effect of SPI concentrations and the endothelial conditions was observed (*p* = 0.034, PE and *p* ≤ 0.001, NA; two-way ANOVA with interaction followed by Bonferroni’s post hoc tests).

## 4. Discussion

Spironolactone (SPI) is a K^+^-sparing diuretic because blocking the effects of aldosterone in the kidney promotes an enhanced K^+^ reabsorption from tubular fluid and increases urinary excretion of Na^+^ [19]. However, some studies have suggested that SPI may be an endocrine disruptor, acting on progesterone and androgen receptors [20]. Therefore, this study aimed to analyze the vascular effects of spironolactone on aorta smooth muscle. For this purpose, rat aorta arteries were used, with both endothelium-intact and -denuded, to investigate the potential disruptive effects of this drug. Several previous studies have examined the effect of spironolactone and its primary metabolite, canrenone, on the contraction and dilation of the isolated aorta [21,22,23]. However, the novel aspects of this work focus on the mode of action of SPI at endothelial level which has never been described. Our main findings revealed that vasodilation induced by SPI on rat aortas is endothelium-dependent, involving NO, and endothelium-independent, blocking Ca^2+^ channels. Prior to the experiments, the effects of spironolactone on the basal tension of the rat aorta were analyzed. The results reveal that SPI did not alter the basal tonus of rat aortas.

In view of these results, the first stage of the experiment was initialized to examine the effects of spironolactone on NA-, PE-, and KCl-contracted aortic rings. The results showed the SPI-induced vasodilation of both endothelium-intact and endothelium-denuded aortic rings. Nevertheless, SPI-induced vasorelaxation was more pronounced in endothelium-intact rings contracted with PE compared to endothelium-denuded rings. A similar effect was observed for KCl contractions, but only at the lowest concentration of SPI (0.001 μmol/L). No differences were found for NA-contractions. To justify these effects, it is important to consider the vascular mode of action (MOA) of the vasoactive agents involved. Noradrenaline (NA) and phenylephrine (PE) are potent α1-adrenoreceptor agonists (Gq-protein coupled) and, therefore, vasoconstrictors of rat aortic smooth muscle. The activation of these receptors stimulates the phospholipase C (PLC)/inositol 1,4,5-trisphosphate (IP3) signaling pathway and increases intracellular Ca^2+^ levels [17,24]. Furthermore, these vasoactive agents may increase extracellular Ca^2+^ influx through voltage- or receptor-operated Ca^2+^ channels [17,24]. On the other hand, NA also activates α2-adrenoreceptors (Gi-protein coupled), which inhibits AC, leading to an increase in intracellular Ca^2+^, similar to α1-adrenoreceptors [25]. As for the activation of β1- and β2-adrenoceptors (Gs-protein coupled), activation by NA may promote vasorelaxation by activating the AC/PKA signaling pathway [26,27]. In the case of KCl contractions, depolarization of the cell membrane by increased K^+^ leads to the activation of LTCC, resulting in an influx of extracellular Ca^2+^ [16].

Given the MOA of the vasoactive agents used, it can be proposed that the effect of SPI involves endothelium-dependent and -independent pathways. The response to NA appears to be endothelium-independent, mediated by the signaling pathways mentioned earlier. Conversely, the effect of PE is enhanced in the presence of endothelium, indicating the contribution of endothelial mediators to the more pronounced vasodilation observed in these arteries. Regarding KCl contractions, it can be inferred that Ca^2+^ channels play a role in the vasodilation induced by SPI, but potential endothelial involvement cannot be ruled out.

Therefore, the next step of this work was to analyze (1) the involvement of Ca^2+^ channels and (2) the involvement of endothelium in SPI-induced vasodilation. The role of Ca^2+^ channels in the vasodilation induced by SPI was investigated using a sensitive organic Ca^2+^ channel blocker, nifedipine (Nif) [16]. The final aim was to understand whether the SPI-induced rat aorta vasodilation could be due to a Ca^2+^ channel blockade, namely LTCC. Our results reveal that the joint application of Nif and SPI produced vasodilation similar to Nif alone in NA- and KCl-contracted rings, supporting our initial hypothesis. The vasodilator mechanism of SPI appears to share the same MOA as nifedipine, i.e., inhibiting LTCC, either in the case of contractions with NA or KCl. In the case of PE, increased vasodilation in endothelium-intact rings indicates endothelial mediators’ possible involvement. This suggests that SPI does not act through the same MOA as nifedipine in the case of PE. Overall, these results confirm the existence of endothelium-dependent and -independent pathways mediate the vasodilatory effect of SPI. These effects depend on the vasoactive agent used, being endothelium-dependent in the case of PE and endothelium-independent in the case of NA and KCl, involving Ca^2+^ channel blockage.

The role of endothelium in the SPI-induced vasodilation was also investigated. According to the literature, the vascular endothelium produces nitric oxide (NO) and prostacyclin (PGI-2), two vasoactive substances involved in endothelium-dependent vasorelaxation [28,29]. The action of these agents is mediated via cyclic nucleotide pathways (NO acts on cGMP signaling, and PGI2 acts on cAMP signaling) [24].

Therefore, to evaluate the role of the endothelium in the vasodilation induced by SPI, two endothelial inhibitors targeting these pathways were chosen: Indo (indomethacin, a non-selective COX inhibitor) and L-NAME (N(ω)-nitro-L-arginine methyl ester, an eNOS inhibitor). The presence of L-NAME resulted in higher tensions in rings contracted with NA and PE compared to endothelium-intact rings, indicating the involvement of NO. However, no significant differences were observed in the presence of indomethacin. These findings align with our previous report on the role of the endothelium in vasodilation induced by a UV filter on the rat aorta [16]. In addition, the data obtained in this study are also in agreement with those previously obtained for metformin in the rat aorta, where it was shown that metformin induces the relaxation of the aorta with and without endothelium. It was also shown that this effect was related to its endocrine-disrupting effect, where metformin was shown to bind to androgen and estrogen receptors by molecular docking [18]. Thus, we can conclude that the data obtained in this study seem to indicate that SPI also has an endocrine-disrupting behavior.

Moreover, the results also show that in the presence of L-NAME inhibitor, SPI-induced vasoconstriction in aortic rings pre-contracted with NA and loses its vasodilatory capacity in rings pre-contracted with PE. L-NAME, a non-selective eNOS inhibitor [30], reduces NO production [31], decreasing vasorelaxation. The significant differences between endothelium-intact and endothelium-denuded rings can be explained by SPI’s loss of vasodilatory capacity in PE-contracted aorta rings for almost all concentrations. Therefore, we can suggest that endothelium-dependent effect of SPI on PE-contractions depends on NO involving the cGMP signaling pathway. On the other hand, the SPI-induced contraction of aorta rings in the presence of L-NAME on NA-contracted rings may be due to the close relationship between NO and endothelin [32]. Indeed, NO production inhibits ET-1 release from the endothelium, whereas ET-1, in turn, greatly inhibits NO-mediated vasodilation [33]. Thus, in the presence of the inhibitor L-NAME, there is a release of ET-1, which will activate the ET_B_ receptor (coupled to the Gq protein) in SMC. Consequently, the PKC/IP3 signaling pathway activates, increasing intracellular Ca^2+^ [34]. In this sense, the observed SPI-induced vasoconstriction in aortic rings pre-contracted with NA and in the presence of L-NAME may be attributed to the activation of the ET_B_ receptor in SMC. On the other hand, in the presence of Indo inhibitor, the results demonstrated a reduction in SPI-induced vasodilation in both NA- and PE-contracted rings. However, these results were only attained for the two highest concentrations. Since indomethacin is a non-selective COX inhibitor, vasodilation decreases [35]. In this sense, our results suggest that SPI-induced vasodilation seems to be only partially dependent on prostacyclin involving the cAMP signaling pathway. However, in the case of NA, this endothelium-dependent effect does not appear sufficient to mediate the vasodilation induced by SPI on rat aortas. The effect of this compound at the endothelial level becomes even more important because there are now other compounds that are also MR antagonists and appear to have an antithrombotic effect. An example of these compounds is eplerenone, a selective steroid mineralocorticoid receptor antagonist, which acts by reducing platelet activation and improving endothelial function by reducing superoxide formation and increasing NO bioavailability, especially in diabetics [36]. On the other hand, there are also non-steroidal MR compounds, which do not have gynecomastia as an adverse effect, with a high affinity (greater than spironolactone and eplerenone) for the mineralocorticoid receptor. One of these compounds is finerenone, which is associated with a lower risk of hyperkalemia, as evidenced by the results of phase I and phase II clinical trial programs [37]. Therefore, these data seem to show that replacing spironolactone with another drug that acts on these receptors, despite being less efficient and more expensive to produce, could be useful in preventing the risk of atherothrombotic and cardiovascular events.

Regarding cell viability assessed by MTT assay, our analysis reveals a decrease in the viability of A7r5 cells upon exposure to the three highest concentrations of SPI used (100, 500, and 1000 μmol/L). Other researchers have reported similar results investigating other EDCs, such as metformin (1000 μmol/L) [18]. However, unlike those studies where the decrease in viability was proportional to the concentration increase, our observations show a non-monotonic effect. Instead, the second-highest concentration (500 μmol/L) caused a more significant decrease in cell proliferation than the highest concentration (1000 μmol/L). This non-monotonic effect aligns with the characteristic behavior of endocrine disruptors [38,39].

In summary, our study revealed that SPI induces vasodilation in the aorta through both endothelium-dependent and -independent mechanisms. Since SPI may promote vasorelaxation in the absence of endothelium, we can conclude that vasodilation occurs directly on the SMC, as Peruskia et al. demonstrated in their evaluation of the steroid testosterone [40]. Consistent with previous findings by Dacquet et al. [41], the endothelium-independent vasodilation induced by SPI seems to involve LTCC, a mechanism also associated with the non-genomic effects of steroid hormones [42]. However, it is noteworthy that this signaling pathway, which plays a crucial role in the effects of endocrine disruptors such as di-(2-ethylhexyl) phthalate (DEHP) [43], tributyltin (TBT) [44], bisphenol A (BPA) [45], and octylmethoxycinnamate (OMC) [16] on rat vasculature, can be impaired by these substances. Conversely, SPI promotes aorta vasodilation through endothelium-dependent mechanisms. This effect was observed specifically in contraction induced by PE, suggesting that SPI-induced vasodilation is dependent on the vasoactive agent used. Interestingly, the effect of NA was relatively similar to PE in arteries with endothelium. These results may suggest that SPI in NA-induced contractions may act not only block α1 and α2 adrenergic receptors but also activate β1 and β2 receptors, promoting vasodilation [45], i.e., a synergistic effect. Worryingly, endothelium-dependent signaling pathways involving cyclic nucleotides have also been associated with the disruptive effects mediated by OMC [16] and BPA [46]. Taken together, our data suggest SPI induces a vasodilation in the rat aorta that is both endothelium-dependent, involving NO, and endothelium-independent (by blocking Ca^2+^ channels). Notably, SPI also exhibited a non-monotonic effect on cell viability, a characteristic pattern observed with EDCs [38,39]. Therefore, given all results observed, it is important to consider the potential for SPI to act as an endocrine disruptor, highlighting the need for further investigations to deepen our understanding of its vascular effects.

## 5. Conclusions

Our results indicated that SPI induces vasodilation in the rat aorta by endothelium-dependent mechanisms involving NO and by endothelium-independent mechanisms blocking Ca^2+^ channels. These results depended on the vasoactive agent used since an endothelium-dependent effect was observed in PE contracts, but in KCl and NA contracts, this effect does not occur. Moreover, in the case of NA, a synergic effect between the α and β adrenergic receptors seems to exist, promoting vasodilation. Concerning the cytotoxicity data, the decrease in cell viability induced by SPI demonstrates a non-monotonic effect characteristic of EDCs. Given all the results obtained, we cannot exclude the possibility that SPI acts as an endocrine disruptor at the vascular level.

## Figures and Tables

**Table 1:** Maximum tensions produced by NA (noradrenaline, 1 μmol/L), PE (phenylephrine, 1 μmol/L), and KCl (potassium chloride, 60 mmol/L) on endothelium-denuded and -intact rings. The values are expressed as tension ± S.D. (mN) of n experiments (in brackets). * (asterisk) represents statistical differences between endothelium-denuded and -intact aorta rings, and ^#^ (cardinal) shows statistical differences between vasoactive agents (*p* < 0.05, two-way ANOVA followed by Bonferroni’s *t*-tests).

Vasoactive Agent	Endothelium-Denuded Rings (mN)	Endothelium-Intact Rings (mN)
NA 1 μmol/L	23.39 ± 6.38 (n = 32) *	14.68 ± 6.35 (n = 22)
PE 1 μmol/L	18.47 ± 7.11 (n = 27) ^#^	15.40 ± 8.94 (n = 28)
KCl 60 mmol/L	22.10 ± 8.27 (n = 31)	22.55 ± 6.84 (n = 28) ^#^

## Data Availability

Data is contained within the article.
